# Bone marrow microenvironment reprogramming in myelodysplastic neoplasms: from pathological mechanisms to targeted therapeutic strategies

**DOI:** 10.3389/fimmu.2026.1726707

**Published:** 2026-02-24

**Authors:** Xiaofei Cong, Xiaohuan Peng, Xixi Chen, Nan Wang, Liansheng Zhang, Lijuan Li

**Affiliations:** 1 Department of Hematology, Second Hospital of Lanzhou University, Lanzhou, Gansu, China; 2 Institute of Hematology, Lanzhou University, Lanzhou, Gansu, China

**Keywords:** immune microenvironment, mesenchymal stem cells, metabolic reprogramming, myelodysplastic neoplasms, targeted therapy

## Abstract

Myelodysplastic Neoplasms (MDS) are a group of clonal hematopoietic malignancies originating from hematopoietic stem cells. Their pathogenesis involves not only genetic abnormalities in hematopoietic cells but is also closely associated with functional dysregulation of the bone marrow microenvironment (BMME). In MDS, both the heterogeneous cellular populations and non-cellular components of the BMME exhibit significant dysfunction. Aberrant BMME components drive the initiation and progression of the disease through complex intercellular interactions. In-depth research into its pathological features and molecular mechanisms is of great significance for developing effective targeted therapeutic strategies. In recent years, novel treatment strategies based on BMME regulation have made significant progress, including immunomodulators, epigenetic regulators, molecularly targeted drugs, and cell therapies, providing new insights for improving the clinical outcomes of MDS patients. This article systematically reviews the pathological features of the BMME in MDS and its key molecular mechanisms in disease development, and discusses the latest clinical research advances in BMME-targeted therapies.

## 1 Introduction

Myelodysplastic Neoplasms (MDS) are a group of clonal hematopoietic stem/progenitor cell disorders characterized by persistent single or multi-lineage cytopenia in peripheral blood, dysplasia in the bone marrow, and a significant risk of transformation to Acute Myeloid Leukemia (AML). The prognosis of MDS patients varies widely, with median survival ranging from several years to just a few months; the transformation rate to AML in high-risk patients can exceed 30% (1). Current treatments—including supportive care, hypomethylating agents, and allogeneic hematopoietic stem cell transplantationudin face challenges, necessitating the development of new therapies. Traditional research has predominantly focused on intrinsic abnormalities of hematopoietic cells. However, a growing body of evidence indicates that the Bone Marrow Microenvironment (BMME)nv dynamic three-dimensional niche composed of heterogeneous cellular populations (mesenchymal stromal cells, osteoblasts/osteoclasts, endothelial cells, adipocytes, neural cells, etc.) and non-cellular components (extracellular matrix, cytokine networks, vascular/neural structures, and metabolites)—plays an indispensable and active role in the pathogenesis, maintenance, clonal evolution, AML transformation, and therapy resistance of MDS (2). Therefore, in-depth analysis of the pathological mechanisms of the BMME in MDS and its interactions with malignant cells is crucial for a comprehensive understanding of the disease. It also provides a critical breakthrough for developing novel BMME-targeted therapeutic strategies, holding significant potential value.

## 2 MDS and microenvironment alterations

### 2.1 Composition of the BMME

**Figure 1:**
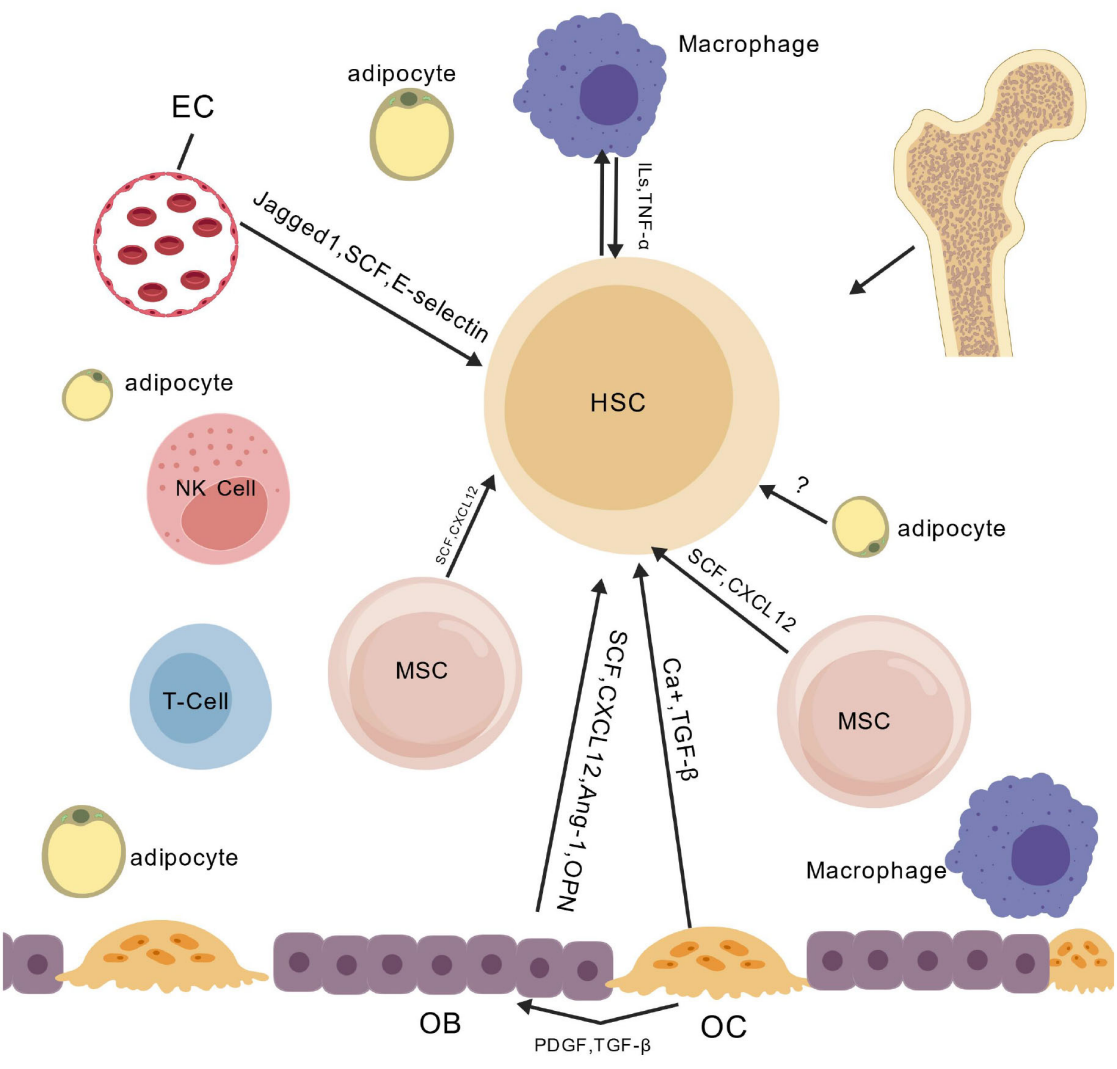
Cellular crosstalk within the bone marrow microenvironment.

The BMME is a highly vascularized three-dimensional structure composed of heterogeneous cellular populations(such as mesenchymal stem cells, osteoblasts, and osteoclasts) and non-cellular components (such as the extracellular matrix and cytokine network). It regulates the homeostasis of Hematopoietic Stem Cells (HSCs) through both physical support and a dynamic signaling network (3–5) (**Figure 1**).

This figure deciphers the molecular mechanisms by which HSC crosstalk with EC, MSC, OB, and OC via Jagged1/SCF/CXCL12-mediated paracrine/cell-contact pathways and IL-1β/TNF-α inflammatory cytokine pathways, collectively regulating HSC self-renewal, differentiation imbalance, and malignant clone expansion in MDS. Abbreviations: EC (Endothelial Cell), MSC (Mesenchymal Stem Cell), HSC (Hematopoietic Stem Cell), OB (Osteoblast), OC (Osteoclast), SCF (Stem Cell Factor), CXCL12 (C-X-C Motif Chemokine Ligand 12), IL-1β (Interleukin-1β), TNF-α (Tumor Necrosis Factor-α).

### 2.2 Pathological alterations of the BMME in MDS

#### 2.2.1 Abnormalities in mesenchymal stem cells

MSCs from MDS patients exhibit significant functional impairments, primarily characterized by accelerated cellular senescence, aberrant differentiation potential, and a compromised ability to maintain BMME homeostasis. Studies have confirmed that MDS-MSCs display marked features of senescence, including elevated β-galactosidase activity, as well as G0/G1 cell cycle arrest and impaired proliferative capacity mediated by the upregulation of the p53/p21 signaling pathway (6, 7). β-galactosidase activity assays revealed higher activity of this enzyme in MSCs from MDS patients compared to healthy controls, visually reflecting the exacerbation of cellular senescence (8). Additionally, epigenetic abnormalities (such as DNMT3A overexpression and DICER1 downregulation (9, 10)) and impaired differentiation potential (inhibition of the Wnt/β-catenin pathway (11, 12)) collectively contribute to the disruption of BMME homeostasis. Research has also found that after endogenous Erythropoietin (EPO) binds to its receptor and activates the JAK2/STAT5 pathway, it inhibits the nuclear translocation of the transcription factor TCF7L2, thereby suppressing osteogenic differentiation (13). MDS-MSCs exhibit prominent genomic instability, which is primarily characterized by the persistent accumulation of DNA damage markers and the frequent occurrence of somatic mutations (14–16). Importantly, this genomic instability represents an intrinsic biological aberration of MDS-MSCs themselves; it can dysregulate the fate of myeloid hematopoietic cells by aberrantly activating the Jagged1-Notch signaling pathway, thereby facilitating disease progression to AML (14–16). Specifically, the genomic instability of MDS-MSCs can induce the abnormal overexpression of the Jagged1 protein. Upon binding to Notch receptors on the surface of myeloid HSCs, this molecule triggers the transcription of downstream target genes (e.g., Hes1, Hey1). This process disrupts the balance between HSCs proliferation and differentiation: on the one hand, it promotes the aberrant proliferation of HSCs by upregulating cyclins (e.g., Cyclin D1); on the other hand, it induces differentiation arrest by inhibiting myeloid differentiation-related transcription factors (e.g., C/EBPα, PU.1). Chronic uncontrolled proliferation coupled with differentiation defects leads to the malignant transformation of HSCs, ultimately driving the progression of MDS to AML (17, 18). It should be emphasized that this transformation process arises from the clonal evolution of hematopoietic cells, rather than the direct transformation of MDS-MSCs into leukemic cells (14).

Furthermore, the Wnt/β-catenin signaling pathway in MDS-MSCs features concurrent decreased activity of the canonical pathway and aberrant activation of non-canonical pathways (19). Meanwhile, the NF-κB signaling pathway is constitutively activated in MDS-MSCs, leading to significantly increased expression levels of inflammatory cytokines IL-6 and TNF-α which exacerbates the local inflammatory response (7, 20). The overexpression of Transforming Growth Factor-beta (TGF-β), synergizing with abnormal microRNA (miRNA) expression profiles, collectively disrupts BMME homeostasis (7, 20).

As core regulatory cells of the local bone marrow immune microenvironment, functional impairments of MDS-MSCs can directly induce immune dysregulation. Under physiological conditions, MSCs maintain bone marrow immune tolerance by secreting immunosuppressive factors such as indoleamine 2,3-dioxygenase (IDO) and prostaglandin E2 (PGE2), which suppress the activation and proliferation of effector T cells and induce the differentiation of regulatory T cells (Treg) (21). However, in MDS patients, MDS-MSCs exhibit defective immunomodulatory functions: on the one hand, their capacity to secrete IDO and PGE2 is markedly diminished, which attenuates the inhibitory effect on effector T cells and triggers local immune activation in the bone marrow; on the other hand, the ability of MDS-MSCs to induce Treg differentiation is impaired, further disrupting immune homeostasis (22, 23). This immune dysregulation sustains a chronic inflammatory microenvironment within the bone marrow, which not only directly impairs normal hematopoiesis, but also accelerates the clonal evolution of malignant HSCs by promoting oxidative stress and DNA damage in these cells (24).

In summary, MDS-MSCs exhibit extensive functional impairments in terms of cellular senescence, epigenetic regulation, differentiation potential, genomic stability, and key signaling pathways (e.g., Wnt, NF-κβ, TGF-β). Moreover, their defective immunomodulatory functions further exacerbate the disruption of the bone marrow microenvironment. The synergistic effects of these multiple abnormalities collectively reveal the central role of MDS-MSCs in the pathogenesis of MDS.

#### 2.2.2 Imbalance between osteoblasts and adipocytes

In patients with MDS, a prominent osteogenic-adipogenic lineage differentiation imbalance exists within the BMME, characterized by decreased osteoblastogenesis accompanied by abnormal adipocyte accumulation. This imbalance represents a core pathological mechanism driving hematopoietic dysfunction, and its upstream drivers arise from the specific regulatory mechanisms governing the differentiation bias of MDS-MSCs.

Suppressed activity of the canonical Wnt/β-catenin signaling pathway serves as the central node triggering lineage differentiation skewing, and this inhibitory effect is synergistically mediated by two mechanisms. First, it is a continuation of the intrinsic defects of MDS-MSCs: overexpression of DNA methyltransferase 3A (DNMT3A) leads to hypermethylation of the promoter regions of key pathway molecules (e.g., Lrp5/6, β-catenin); downregulation of DICER1 disrupts the expression profile of miRNAs that regulate the pathway; and cellular senescence mediated by p53/p21 pathway activation. These three factors jointly impair the transcriptional activity of the pathway and directly impede osteogenic differentiation (25). Second, it is the paracrine reverse regulation by malignant hematopoietic clones: EPO secreted by malignant hematopoietic stem/progenitor cells (HSPCs) can activate the JAK2/STAT5 pathway in MSCs, blocking pathway signal transduction by inhibiting the nuclear translocation of transcription factor TCF7L2. Meanwhile, the released inflammatory factors (e.g., IL-6, TNF-α, can activate the NF-κβ pathway, upregulating the expression of Dickkopf-related protein 1 (DKK1), an inhibitor of the Wnt pathway, thereby further suppressing osteogenic differentiation and promoting adipogenic differentiation (26, 27).

Suppressed activity of the Wnt/β-catenin pathway impairs the osteogenic differentiation of mesenchymal stem cells (MSCs), which in turn weakens their physical anchoring of hematopoietic stem cells (HSCs) and the provision of critical supportive signals (e.g., Notch ligands, Angiopoietin-1), ultimately compromising the homing, quiescence maintenance and differentiation of HSCs (28).Concurrently, aberrant expansion of adipocytes suppresses hematopoiesis through multiple pathways: Firstly, metabolic toxicity, involving excessive secretion of free fatty acids (FFAs) and reactive oxygen species (ROS), directly damages HSC mitochondrial function, triggering energy metabolism disorders and DNA damage (28–30). Secondly, overexpression of inhibitory factors occurs, such as aberrantly high expression of the Wnt pathway inhibitor Dickkopf-related protein 1 (DKK1), further exacerbating osteogenic impairment and Wnt inhibition, the adipogenesis master regulator PPARγ (directly inhibiting HSC proliferation and differentiation), and the inflammation-related factor leptin. The abnormally expanded adipocytes also secrete TGF-β inducing HSC cell cycle arrest and apoptosis (28–30).

Intervention strategies targeting this imbalance primarily focus on activating the Wnt/β-catenin pathway to promote osteogenesis/inhibit adipogenesis: For example, directly activating the Wnt pathway using GSK3β inhibitors (e.g., lithium chloride, CHIR99021) to stabilize β-catenin) or developing Wnt agonists to drive MSC osteogenic differentiation. Another approach involves antagonizing key inhibitory factors, such as using anti-DKK1 antibodies (e.g., DKN-01) to neutralize its inhibitory effect, or employing PPARγ antagonists/modulators (SPPARγMs) to moderately inhibit adipogenesis. Neutralizing TGF-β via antibodies, receptor traps, or kinase inhibitors (e.g., Galunisertib), can block its pro-apoptotic effects. Finally, combination therapy, integrating the aforementioned BMME-targeting drugs with hypomethylating agents, can simultaneously suppress the malignant clone and repair the microenvironment, thereby more effectively improving ineffective hematopoiesis (31, 32).

#### 2.2.3 Abnormal vascular microenvironment

In the pathological process of MDS, the structural and functional abnormalities of the bone marrow vascular system interact closely with MDS-HSPCs, directly affecting disease progression. Studies have shown that MDS-HSPCs can secrete vascular endothelial growth factor (VEGF), which binds to receptors on the surface of vascular endothelial cells to activate downstream signaling pathways and drive abnormal angiogenesis in the bone marrow (28, 33). The newly formed abnormal blood vessels are characterized by structural disorder and increased permeability. MDS-HSPCs can adhere to vascular endothelial cells via surface integrins and secrete matrix metalloproteinases to degrade the vascular basement membrane, thereby promoting their own infiltration and dissemination within the bone marrow microenvironment. Insufficient blood perfusion caused by abnormal blood vessels directly induces hypoxia in the BMME, which further upregulates the expression of hypoxia-inducible factor-1α (HIF-1α), enhances VEGF secretion, regulates the metabolic pattern of MDS-HSPCs, accelerates the enhancement of their malignant phenotype, recruits immune cells to exacerbate local inflammatory responses, and inhibits anti-tumor immune function (28, 33). In addition, vascular endothelial cells and MDS-HSPCs can achieve cross-activation through the Notch signaling pathway, dually promoting abnormal angiogenesis in the bone marrow and the invasive capacity of MDS-HSPCs (34). In-depth clarification of the above MDS-specific vascular-related regulatory mechanisms may provide new strategic directions for the targeted therapy of MDS.

#### 2.2.4 Dysregulation of the inflammatory and immune microenvironment

The bone marrow immune microenvironment in MDS patients exhibits significant disruption, characterized by extensive quantitative and functional abnormalities in various immune cell populations. Complex synergistic and antagonistic interactions exist among these immune cells, collectively driving disease progression and immune escape. Regarding T cells, there is an expansion of the CD8+CD57+CD28- senescent subset within CD8+ T cells, accompanied by upregulated NKG2D/CD244 expression and downregulated CCR7/CD62L expression (35–37); these activated senescent CD8+ T cells can target not only malignant clone cells but also normal hematopoietic progenitor cells, suppressing normal hematopoiesis (38). Concurrently, a reduction in CD4+ T cell numbers leads to a decreased CD4/CD8 ratio (23). Imbalance also exists among T cell functional subsets: low-risk patients are characterized by increased Th17 cells and decreased Tregs, whereas high-risk patients show a particularly pronounced Th17/Treg imbalance, accompanied by increased Tregs and decreased CD8+ T cells and Natural Killer (NK) cells (39–42). T cell exhaustion often emerges in advanced stages (43). Functional defects in macrophages and NK cells act synergistically. Macrophages exhibit impaired phagocytic capacity in high-risk stages due to CD47 overexpression and intrinsic dysfunction (44–48), while NK cells fail to effectively clear MDS cells due to reduced cytotoxicity and decreased expression of activating receptors (49–51). Together, this weakens the body’s surveillance capability against MDS cells. Dendritic cells show numerical and functional deficiencies, leading to insufficient antigen presentation and ineffective T cell activation (52, 53). Myeloid-Derived Suppressor Cells (MDSCs) promote T cell exhaustion via CD33-dependent mechanisms and the galectin-9 pathway, forming an immunosuppressive loop (54). Additionally, the overall BMME in MDS exhibits a chronic inflammatory state. Elevated levels of pro-inflammatory cytokines not only induce apoptosis of hematopoietic cells but may also promote genomic instability (55), further aiding MDS cells in evading immune attack.

Immunotherapy targeting this dysregulation brings new hope for MDS. Immune modulators targeting T cell senescence, correcting Th17/Treg imbalance, and drugs activating NK cell and macrophage function hold promise for reshaping anti-tumor immunity. However, treatment faces multiple challenges: the stage-specific heterogeneity of immune cell function necessitates precise, stratified intervention; the complexity of the immunosuppressive network means single-target therapies easily trigger compensatory escape mechanisms; furthermore, normal hematopoietic cells are susceptible to immune attack, requiring a balance between efficacy and myelosuppression risk. Therefore, comprehensive strategies combining immune modulation, targeting tumor escape mechanisms, and supporting hematopoiesis may become key to breaking through the therapeutic dilemma in MDS.

#### 2.2.5 Alterations in the metabolic microenvironment

The pathogenesis of MDS is closely associated with the dysregulation of metabolic homeostasis in the BMME, which drives disease progression by regulating the function of HSCs. Under chronic hypoxic stress, HIF-1α is stably expressed in bone marrow HSCs, MSCs, and bone marrow stromal cells of MDS patients. On one hand, it upregulates proinflammatory factors such as IL-6 and TNF-α (56); on the other hand, it activates the aerobic glycolysis pathway, presenting metabolic characteristics of enhanced glycolysis and suppressed oxidative phosphorylation (OXPHOS) (57). OXPHOS inhibition can induce ROS accumulation through electron transport chain (ETC) dysfunction, specifically manifested as blocked ETC electron transfer leading to increased electron leakage, which combines with oxygen in the mitochondrial matrix to generate ROS such as superoxide anions. Meanwhile, elevated mitochondrial membrane potential further exacerbates electron leakage, accompanied by reduced ATP synthesis that impairs the activity of antioxidant enzymes and weakens ROS scavenging capacity, ultimately resulting in abnormal ROS accumulation. These changes not only promote the proliferation of malignant HSCs but also induce chemotherapeutic drug resistance, accelerating disease progression (58, 59). Metabolic disorders and the inflammatory microenvironment form a positive feedback loop. Stable HIF-1α expression in the aforementioned cells not only enhances glycolysis but also directly impairs the genomic stability of HSCs through ROS accumulation, while inhibiting the proliferation and differentiation of normal HSCs, further exacerbating hematopoietic dysfunction (60).

Dyslipid metabolism is mainly characterized by enhanced FAO in malignant HSCs and bone marrow stromal cells, which forms a synergistic effect with autophagy activation (61). The fatty acids involved in FAO here are mainly derived from intracellular lipids degraded by autophagy: autophagy degrades intracellular lipids to generate free fatty acids, which are transported to mitochondria via CPT1A highly expressed on the cell membrane of malignant HSCs for FAO, selectively meeting the energy demand of malignant clonal cells and promoting their proliferation. Meanwhile, abnormal activation of the de novo phospholipid synthesis pathway mediated by phosphatidylcholine synthase/phosphatidylethanolamine-N-methyltransferase in bone marrow stromal cells, together with dysregulated phospholipid metabolic pathways in malignant HSCs, collectively leads to elevated levels of phospholipid metabolites (such as phosphatidylcholine and phosphatidylethanolamine), which is significantly associated with an increased risk of MDS progression to AML (62, 63).

Iron homeostasis imbalance stems from ineffective hematopoiesis and transfusion-related iron overload, mainly affecting bone marrow monocytes, macrophages, and malignant HSCs (64): the decreased ATP/AMP ratio in these cells reflects OXPHOS uncoupling, thereby triggering ROS accumulation and genomic damage. ROS not only directly destroys HSC genomic integrity but also synergizes with HIF-1α signaling to amplify glycolytic dependence, further inhibiting normal HSC function and promoting malignant clone expansion (58, 64).

Mitochondrial dysfunction mainly occurs in HSCs and bone marrow stromal cells of MDS patients, resulting in the loss of cellular metabolic plasticity and manifested as persistent aerobic glycolysis. Abnormalities of key mitochondrial proteins are also concentrated in these cells; for example, RISP deficiency inhibits the function of mitochondrial respiratory chain complex III, and PTPMT1 deficiency impairs mitochondrial phospholipid metabolism and inhibits mitochondrial fusion and fission, both leading to HSC differentiation arrest and failure to normally differentiate into mature blood cells (65, 66).

In response to the above metabolic abnormalities, various targeted intervention strategies have been explored, and their intervention mechanisms are closely related to BMME regulation: ① Targeting glycolysis: HK2 inhibitors can block key steps of glycolysis in HSCs and bone marrow stromal cells, inhibiting energy supply for malignant clones; PDK1 inhibitors relieve the inhibitory effect on OXPHOS by inhibiting PDK1 activity in HSCs and BMSCs, reverse the glycolytic-dominant phenotype, restore metabolic plasticity of normal HSCs, and weaken the support of bone marrow stromal cells for malignant clones (67, 68). ② Targeting FAO: The CPT1A inhibitor etomoxir can inhibit CPT1A-mediated mitochondrial transport of free fatty acids in malignant HSCs; combined with autophagy inhibitors, it can block autophagy-dependent lipid degradation in bone marrow stromal cells and malignant HSCs, dually cutting off the fatty acid energy supply pathway and inhibiting malignant clone proliferation (69). ③ Targeting iron metabolism: The iron chelator deferasirox can reduce ROS accumulation induced by iron overload in monocytes, macrophages, and HSCs, alleviate oxidative stress-induced damage to normal HSCs, and inhibit the secretion of proinflammatory factors by bone marrow stromal cells, improving BMME support function (70).

These metabolic enzymes (HK2, PDK1, CPT1A), transporters (CPT1A), and mitochondrial proteins (PTPMT1, RISP) serve as potential therapeutic targets; the progress in related drug research and development provides new directions for intervening in MDS disease progression by targeting metabolic pathways. The multi-dimensional metabolic disorders in multiple cell populations such as HSCs and bone marrow stromal cells jointly constitute the BMME basis supporting malignant clone proliferation.

## 3 Therapeutic strategies targeting BMME regulation

In recent years, aberrant remodeling of the BMME has emerged as a core mechanism driving hematopoietic failure, clonal proliferation, and disease progression in MDS. Dysregulation of BMME components (such as immune cell imbalance and stromal cell dysfunction) interacts reciprocally with abnormal signaling pathways, forming a pro-tumorigenic pathological microenvironment. Novel therapeutic strategies based on precise regulation of BMME have achieved continuous breakthroughs, including immunomodulators, epigenetic regulators, molecular targeted drugs, and cellular therapies. By correcting the pathological state of BMME and restoring hematopoietic homeostasis, these strategies provide critical new insights for improving the clinical prognosis of MDS patients. The following review focuses on the targeted regulatory mechanisms of various strategies on BMME and the research progress of related drugs.

### 3.1 Epigenetic modulating drugs

Gain-of-function mutations in isocitrate dehydrogenase 1/2 (IDH1/2) are key drivers of epigenetic dysregulation in MDS. These mutations aberrantly produce 2-hydroxyglutarate (2-HG), inducing excessive DNA and histone methylation, which not only directly impairs hematopoietic stem cell function but also accelerates disease progression by remodeling the immunosuppressive phenotype of the BMME (71). The core value of such inhibitors lies in reversing epigenetic abnormalities to simultaneously restore the hematopoietic supportive function and immune regulatory balance of the BMME, rather than merely killing MDS cells.

Ivosidenib, a selective IDH1 inhibitor, reduces 2-HG levels by blocking mutant IDH1 activity. On one hand, it induces histone demethylation to promote the differentiation of abnormal hematopoietic cells; on the other hand, it significantly inhibits the recruitment and immunosuppressive function of MDSCs in the BMME, reduces their cytotoxic inhibition on CD8^+^ T cells, and enhances the ability of bone marrow stromal cells to secrete hematopoietic growth factors (e.g., G-CSF, EPO), thereby restoring the hematopoietic supportive properties of the BMME (72). Clinical data show that in the treatment of relapsed/refractory MDS patients, the median overall survival reaches 35.7 months with an overall response rate of 83% (72). The therapeutic benefit is directly correlated with the degree of epigenetic repair in the BMME. Monotherapy exhibits favorable safety profile, with common adverse reactions such as rash and diarrhea mostly mild to moderate and well-tolerated by patients (72). The drug resistance mechanisms mainly involve the activation of compensatory signaling pathways such as PI3K/Akt in the BMME, leading to re-enrichment of MDSCs, which necessitates combination with targeted pathway inhibitors to optimize efficacy.

When olutasidenib is combined with azacitidine for the treatment of IDH1-mutated MDS/AML patients, the overall response rate is increased to 86%, and there is a synergistic effect between the two drugs in BMME regulation: azacitidine improves the hematopoietic supportive function of stromal cells through demethylation, while olutasidenib enhances the reversal of the immunosuppressive microenvironment, further reducing PMN-MDSCs infiltration and strengthening anti-tumor immune response. However, combination therapy may exacerbate the proliferation inhibition of hematopoietic cells in the BMME and increase hematological toxicities such as myelosuppression, which requires dose optimization to balance efficacy and safety (73). Currently, there is limited research on its drug resistance characteristics, and more clinical observations are needed to verify the impact on the expression of BMME resistance-related molecules.

For patients with IDH2 mutations, enasidenib combined with azacitidine yields a median overall survival of 26 months and an overall response rate of 74% (74). Its core regulatory effect on the BMME is to inhibit the excessive activation of the NF-κB signaling pathway in bone marrow stromal cells, reduce the secretion of pro-inflammatory cytokines (IL-6, TNF-α), decrease the pro-clonal proliferative activity of the BMME, and simultaneously improve the adhesion function between hematopoietic stem cells and stromal cells to restore the normal hematopoietic niche (75). In terms of safety, elevated bilirubin is the main side effect (74), which can be managed with symptomatic treatments. The drug resistance characteristics are similar to those of IDH1 inhibitors, both associated with re-abnormality of the microenvironment caused by compensatory activation of signaling pathways in the BMME.

In the exploration of combination therapy, the synergistic core of IDH inhibitors and hypomethylating agents lies in the dual repair of BMME epigenetic dysregulation and hematopoietic homeostasis (73). Future clinical trials will focus on the combination of IDH inhibitors with immune checkpoint inhibitors, which achieves dual regulation of the BMME through “epigenetic repair + immune activation”, further eliminating residual clones, delaying disease progression, and bringing more durable BMME function improvement and survival benefits to MDS patients.

### 3.2 Drugs targeting RNA splicing abnormalities

Aberrant RNA splicing is one of the core molecular features of MDS, which not only causes abnormal expression of hematopoiesis-related genes but also exacerbates bone marrow stromal cell dysfunction and the formation of a hematopoietic-suppressive microenvironment by regulating key signaling pathways in the BMME (e.g., TGF-β/Smad, SF3b complex-mediated pathways). Drugs targeting splicing abnormalities can simultaneously improve hematopoietic cell function and the pathological state of the BMME by correcting molecular splicing defects.

Luspatercept, an erythroid maturation agent, has a clear targeted regulatory mechanism on the BMME: it selectively binds to overactivated TGF-β superfamily ligands (e.g., GDF11, BMP6) in the BMME, blocking abnormally enhanced Smad2/3 signaling (76). On one hand, it directly promotes the maturation of erythroid precursor cells and improves ineffective hematopoiesis; on the other hand, it inhibits the secretion of pro-apoptotic factors (e.g., FasL) by bone marrow stromal cells, reduces hematopoietic stem cell apoptosis, and simultaneously decreases pro-inflammatory cytokine levels in the BMME to alleviate chronic inflammatory damage to the microenvironment (77). Clinically, it can significantly improve anemia symptoms and transfusion dependence in lower-risk MDS patients, and its efficacy is positively correlated with the degree of TGF-β signaling pathway inhibition in the BMME. It has been included in the first-line recommendations of NCCN and CSCO guidelines, becoming a key drug for improving the prognosis of lower-risk MDS by regulating BMME signaling pathways (76).

H3B-8800 is an ATP-dependent 17S U2 snRNP complex inhibitor that corrects aberrant RNA splicing in MDS by interfering with SF3b spliceosome binding (78). Its regulatory effect on the BMME is mainly reflected in reducing the expression of aberrantly spliced pro-tumor factors (e.g., MMP9, VEGF) in bone marrow stromal cells, inhibiting excessive angiogenesis and stromal cell fibrosis in the BMME, and restoring the expression of normally spliced immune regulatory molecules (e.g., PD-L1), laying a foundation for subsequent combination immunotherapy (79). But long-term safety and the differences in BMME regulation among MDS patients with different risk stratifications still need further observation.

In the exploration of combination therapy, the combination of such splicing inhibitors with hypomethylating agents can synergistically improve the hematopoietic-suppressive and pro-tumor properties of the BMME through “splicing repair + epigenetic correction”, further improving the response rate. However, attention should be paid to the excessive inhibition of hematopoietic cell proliferation in the BMME by combination therapy, and clinical studies are needed to optimize dosage regimens to balance the efficacy of BMME repair and hematopoietic toxicity.

### 3.3 Drugs targeting anti-apoptotic proteins

Excessive activation of anti-apoptotic signaling pathways (e.g., high BCL-2 expression) in the BMME is a key mechanism contributing to apoptotic evasion of abnormal clonal cells and suppression of normal hematopoietic cell survival in MDS (80). BCL-2 is not only highly expressed in MDS clones but also abnormally upregulated in stromal cells and MDSCs within the BMME, maintaining the pathological homeostasis of the microenvironment by inhibiting cell apoptosis (80). Thus, BCL-2-targeted inhibitors can act synergistically on both tumor clones and the BMME to achieve dual regulation (81).

Venetoclax, a selective BCL-2 inhibitor, exerts its core regulatory effects on the BMME by inducing BCL-2-dependent apoptosis. It eliminates abnormally proliferative stromal cells and MDSCs in the BMME, reduces the proportion of immunosuppressive cells, and restores the anti-tumor activity of CD8^+^ T cells and NK cells. Meanwhile, it downregulates the compensatory expression of anti-apoptotic factors (e.g., BCL-XL) in the BMME, disrupts the pro-survival signaling network of the microenvironment, improves abnormal adhesion between hematopoietic stem cells and the BMME, and promotes the engraftment of normal hematopoietic cells (82, 83).

### 3.4 Other targeted drugs

#### 3.4.1 Optimized chemotherapeutic preparations

As a nanoliposomal complex of cytarabine and daunorubicin, CPX-351 exhibits core advantages over traditional chemotherapy by enhancing targeted enrichment of drugs in bone marrow tissues. While reducing damage to normal tissues, it strengthens the clearance of abnormal components in the BMME: it can significantly eliminate abnormally proliferative stromal cells and vascular endothelial cells in the BMME, inhibit excessive bone marrow angiogenesis, reduce the pro-clonal proliferative activity of the BMME, and simultaneously decrease MDSC infiltration to alleviate the immunosuppressive microenvironment (84, 85). Clinical data show that its overall response rate and complete response rate in the treatment of high-risk MDS reach 87% and 52%, respectively (85). The therapeutic benefits are directly associated with reduced vascular density and decreased proportion of immunosuppressive cells in the BMME, providing an optimized chemotherapeutic option for high-risk MDS patients that balances BMME repair and tumor clearance (86).

#### 3.4.2 NEDD8-activating enzyme inhibitors

Pevonedistat, a selective NEDD8-activating enzyme inhibitor, blocks the ubiquitination modification pathway to not only inhibit the proliferation of abnormal MDS clones but also precisely regulate the NF-κB signaling pathway in the BMME. It reduces the secretion of pro-inflammatory cytokines (IL-6, IL-1β) and pro-angiogenic factors (VEGF) by bone marrow stromal cells and MDSCs, inhibits chronic inflammation and angiogenesis in the BMME, and enhances the self-renewal capacity and differentiation potential of hematopoietic stem cells (87). Clinical studies have confirmed that when combined with azacitidine in the treatment of high-risk MDS patients, its median event-free survival is significantly superior to azacitidine monotherapy (88). This advantage stems from the synergistic regulation of the BMME by the two drugs: azacitidine improves epigenetic abnormalities, while Pevonedistat inhibits inflammatory signaling pathways, jointly reversing the pro-tumor phenotype of the BMME (88).

#### 3.4.3 Immune checkpoint inhibitors

Aberrant activation of the programmed cell death protein 1 (PD-1)/programmed death-ligand 1 (PD-L1) pathway in the BMME of MDS patients is a core link in the formation of the immunosuppressive microenvironment. PD-L1 is highly expressed on the surface of MDSCs, tumor cells, and stromal cells in the BMME; it induces T cell exhaustion by binding to PD-1 on T cells, impairs anti-tumor immune responses, and promotes MDSC recruitment, forming an immunosuppressive loop (89). The core value of PD-1 inhibitors is to break the BMME immunosuppressive network and restore anti-tumor immune function (90).

Pembrolizumab shows more significant efficacy in MDS patients with high PD-L1 expression (91). It specifically binds to PD-1 on T cells, blocks PD-1/PD-L1 interaction, reverses T cell exhaustion in the BMME, enhances the cytotoxic activity of CD8^+^ T cells against abnormal hematopoietic clones, and simultaneously reduces the immunosuppressive function of MDSCs, converting the BMME from an “immunosuppressive” to an “immune-activated” state (92). Clinical data show that the overall response rate of pembrolizumab combined with azacitidine in the treatment of newly diagnosed high-risk MDS patients reaches 76% (93). The synergistic mechanism is that azacitidine upregulates PD-L1 expression in MDS cells and immune cells within the BMME through demethylation, enhancing the immune activation effect of pembrolizumab. However, attention should be paid to immune-related adverse reactions (such as immune pneumonia and thyroid dysfunction), whose occurrence is associated with excessive immune activation in the BMME, requiring close monitoring and timely intervention (91). In addition, the combination of PD-1 inhibitors with novel immune-targeted drugs such as CD300ld inhibitors can further enhance the clearance of PMN-MDSCs in the BMME and improve immune regulatory efficacy, which has become an important research direction in immunotherapy for high-risk MDS (94).

## 4 Summary and outlook

This article has systematically elaborated on the pathological features of the BMME in MDS and its key molecular mechanisms in disease pathogenesis and progression, emphasizing the significant value of BMME-targeted therapy in improving the clinical outcomes of MDS patients. The pathogenesis of MDS involves not only genetic mutations in hematopoietic stem cells but also the active role of BMME abnormalities in driving disease progression through mechanisms such as inflammatory responses, immune dysregulation, and metabolic reprogramming. Therapeutic strategies targeting the BMME offer potential targets for MDS treatment, and their combination with traditional therapies holds promise for improving patient prognosis. Future research should leverage single-cell sequencing and spatial transcriptomics to decipher the spatiotemporal interactions between the microenvironment and malignant clones, and explore novel combination strategies targeting metabolic-immune cross-talk pathways (e.g., IDO1/HIF-1α to advance the development of precision medicine for MDS.
